# resistancebank.org, an open-access repository for surveys of antimicrobial resistance in animals

**DOI:** 10.1038/s41597-021-00978-9

**Published:** 2021-07-22

**Authors:** Nicola G. Criscuolo, João Pires, Cheng Zhao, Thomas P. Van Boeckel

**Affiliations:** 1 Institute for Environmental Decisions, ETH Zürich, Zurich, Switzerland; 2 Center for Disease Dynamics, Economics and Policy, New Delhi, India

**Keywords:** Epidemiology, Antimicrobial resistance, Bacterial infection, Health policy

## Abstract

Antimicrobial resistance (AMR) is a growing threat to the health of humans and animals that requires global actions. In high-income countries, surveillance systems helped inform policies to curb AMR in animals. In low- and middle-income countries (LMICs), demand for meat is rising, and developing policies against AMR is urgent. However, surveillance of AMR is at best nascent, and the current evidence base to inform policymakers is geographically heterogeneous. We present *resistancebank.org*, an online platform that centralizes information on AMR in animals from 1,285 surveys from LMICs. Surveys were conducted between 2000 and 2019 and include 22,403 resistance rates for pathogens isolated from chickens, cattle, sheep, and pigs. The platform is built as a *shiny* application that provides access to individual surveys, country-level reports, and maps of AMR at 10 × 10 kilometers resolution. The platform is accessed via any internet browser and enables users to upload surveys to strengthen a global database. *resistancebank.org* aims to be a focal point for sharing AMR data in LMICs and to help international funders prioritize their actions.

## Introduction

Antimicrobials are essential drugs that have helped considerably reducing infectious diseases mortality. However, in recent years, their overuse in human medicine and animal production^1–4^ has caused a rise in antimicrobial resistance^5–7^ (AMR). Globally, 73% of all antimicrobials are used in animals to prevent and treat infections^8^, but also to improve weight gain and productivity on farms^9^. The rise of antimicrobial use and resistance in animals is a growing concern for the future of animal health, and for the livelihood of billions of people who rely on animals for subsistence^10,11^. In addition, in recent years a growing body of evidence suggested that antimicrobial-resistant bacteria can be transferred between animals to humans^12–16^, and cause drug-resistant infections in humans^17^. As for other infectious diseases of global importance^18–20^, the rise of AMR in animals is a health challenge that requires close monitoring to coordinate international actions.

In high-income countries, trends in AMR in animals are monitored via systematic surveillance^21^ by organizations such as the European Food Safety Authority (EFSA) in Europe, the National Antimicrobial Resistance Monitoring System for Enteric Bacteria (NARMS) in the United States, or the Canadian Integrated Program for Antimicrobial Resistance Surveillance (CIPARS) in Canada^22^. However, in low- and middle-income countries (LMICs), where demand for meat (and antimicrobials) is rising rapidly^2,11^, systematic surveillance systems remain largely absent^23^. International actions to set-up or scale-up surveillance systems in LMICs have been initiated^24,25^. However, these may take years to be fully operational to inform policymakers. In the short term, efforts to target investments in LMICs against AMR could be informed by point-prevalence surveys^26^ (PPS). Hundreds of PPS on foodborne pathogens are conducted each year across LMICs. In the absence of systematic surveillance systems, these could be used to document trends in AMR in food animals. In 2019, PPS that were initially scattered across the veterinary scientific literature were systematically reviewed to produce a first global map of AMR in food animals at a sub-national level^26^. In addition, the findings of systematic reviews of PPS could be used to identify hotspots of resistance in animals in LMICs where stewardship efforts should be focused, or to identify areas poorly surveyed, where recruiting local epidemiologists may help improve the assessments of the AMR situation.

However, systematic surveys are time-consuming, need to be repeated frequently, and may require access to publications outside of the public domain. In addition, valuable information on AMR may be missed in systematic reviews due to i) linguistic barriers, ii) expensive publishing fees of international journals for researchers in LMICs, and iii) data availability restrictions from industry-program sponsor or governmental monitoring programs. An open-access platform for reporting results of PPS on AMR in real-time could help overcome these limitations and empower local communities of researchers. A platform designed with an intuitive interface may also encourage data sharing in the AMR community. This would not only improve the circulation of knowledge between researchers but also strengthen estimates of the AMR burden, and provide up-to-date information to policymakers who allocate resources for intervention.

Online platforms have enabled data sharing in multiple scientific fields^27–30^. In epidemiology, they are used, amongst others, to integrate translational medicine data^31^, exchange datasets of high-risk tumors^32^, or disseminate estimates of the burden of malaria^33–35^. In AMR epidemiology, online platforms have been introduced to report drug-resistant infections in humans^33^. Thus far a comparable tool is missing for reporting AMR levels in animals. The development of such a tool to encourage data aggregation and visualization has been recognized as a priority by international donors^36,37^ and organizations^38,39^.

Here, we introduce *resistancebank.org*, an online platform for surveys and maps of AMR in animals. First, we present a database of PPS reporting AMR rates globally. Second, we introduce local indicators of AMR burden available for download: maps and country-level reports. Third, we provide a step-by-step guide of the User Interface (UI), as seen by the visitors of the platform to upload their data in *resistancebank.org*.

## Methods

### Database

We conducted a systematic literature search in January 2019 and extracted information on resistance rates from 1,285 PPS. The search for PPS on AMR in food animals from LMICs was conducted in three bibliographic databases: PubMed, Scopus, and Web of Science. We targeted four indicator bacteria recommended by the World Health Organization’s Advisory Group on Integrated Surveillance of Antimicrobial Resistance (AGISAR). Titles and abstracts were deduplicated and screened for PPS. Books, meta-analysis, reviews, and PPS reporting on sick animals were excluded, according to the AGISAR guidelines^40^. In addition, we included data available in paper journals, Ph.D. and MSc thesis, and conference proceedings after field visits to five veterinary schools in India. All relevant data to AMR surveillance were screened across all manuscripts, including sampling size, animal hosts, bacterial species, sampling latitude and longitude. The resistance rates from antimicrobial susceptibility testing (AST) were aggregated by individual location/host/bacteria combinations. For each study, all tested antimicrobials and the number of isolates included in each assay were recorded. For the geospatial analysis, only antimicrobials recommended by the AGISAR were used. A description of the database variables reported in Table 1 can be found on *resistancebank.org*. For a detailed explanation of the methods used for the literature search see the Supplementary Materials of Van Boeckel & Pires, 2019^26^.

**Table 1:** Metadata of the variables available in *resistancebank.org*.

DOI	Digital object identifier or PubMed ID
Author	Name of the first author
ISO3	Country identifier of the International Organization of Standardization.
YCoord, XCoord	Latitude and longitude of the PPS in decimal degrees
StartDate, EndDate	Starting and ending date of field samplings (day/month/year)
Species	Animal host species
SampleOrigin	Type of biological sample used to isolate bacteria from
Method	Experimental Methodology for the AST
Pathogens	Bacterial species
Strain	Bacterial subtype
Nsamples	Number of samples collected
Prev	Pathogen prevalence
NIsolates	Number of isolates used for AST
Class	Classification of antimicrobials based on their chemical structure
Compound	Antibiotic molecule
ATC.Code	Anatomical Therapeutic Chemical identifier of the compound
Rescom	Percentage of isolates resistant for a given drug-pathogen combination
Concg	Concentration/amount of antimicrobial used for AST
Guidelines	Guideline used for antimicrobial AST (document and year)
Breakpoint	Resistance breakpoint used for interpreting AST results
Remarks	Comments relative to the publication.

**Fig. 1:**
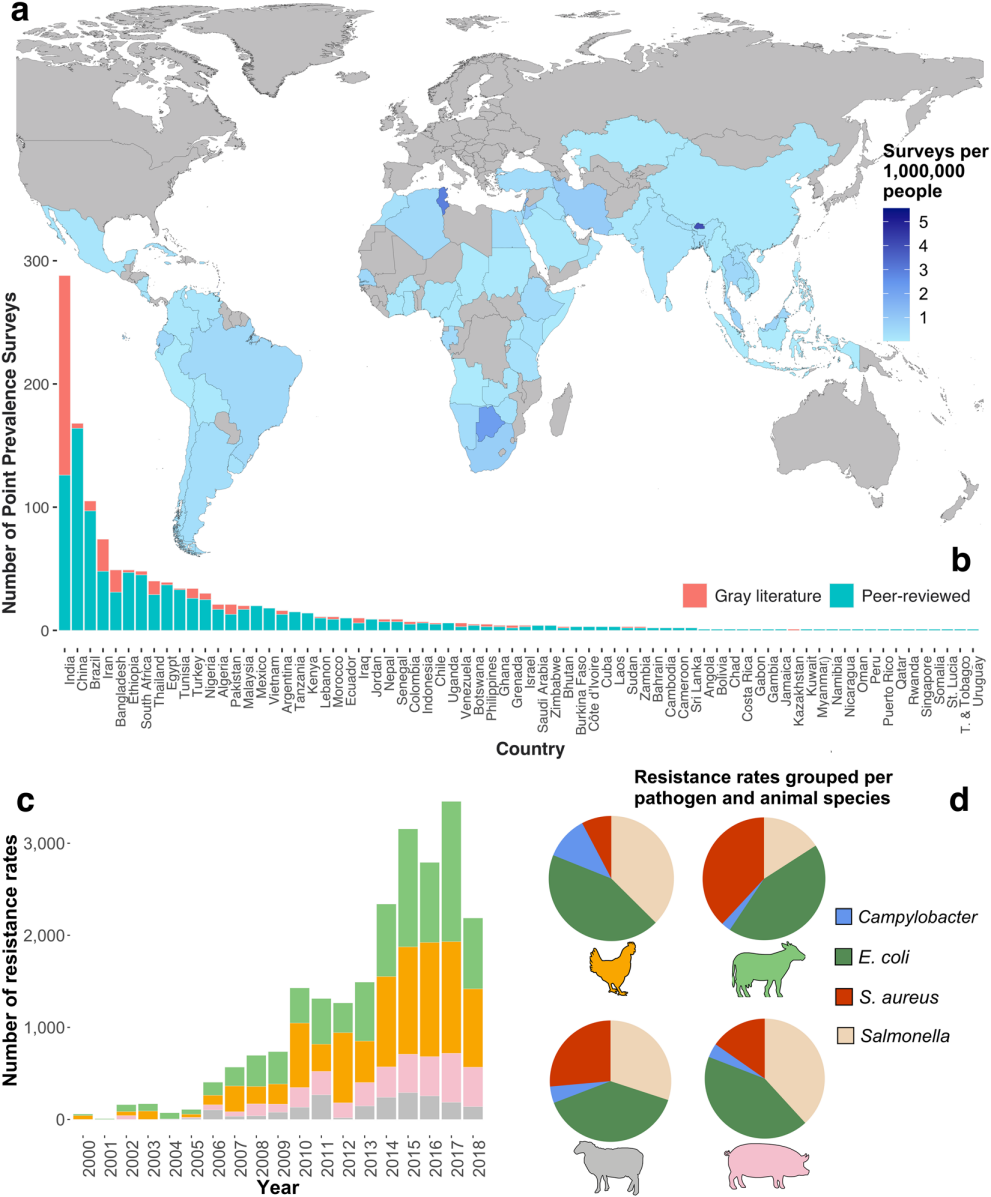
Metadata of *resistancebank.org*. (**a**) Number of surveys per 1 million people in LMICs. (**b**) Point-prevalence surveys grouped by country and literature source (peer-reviewed vs gray literature). (**c**) Number of resistance rates grouped by year and animal species. (**d**) Resistance rates grouped by pathogen and animal species.

The database contains 22,403 resistance rates (*n*) extracted from PPS conducted on foodborne pathogens in LMICs between 2000 to 2019 (Fig. 1). The pathogens isolated for AST include *Escherichia coli* (*n* = 9,206, 41.09%), non-typhoidal *Salmonella* spp. (*n* = 7,080, 31.60%), *Staphylococcus aureus* (*n* = 4,828, 21.55%), and *Campylobacter* spp. (*n* = 1,290, 5.76%). The PPS were conducted in 72 out of the 135 countries classified as LMICs by The World Bank^41^. Overall, 61.4% of all PPS listed on *resistancebank.org* were conducted in Asia, 24.1% in Africa, and 14.5% in Central and South America (Fig. 1); 49.41% of PPS were collected in India, China, Brazil, and Iran. Across all studies, bacteria were isolated from poultry (38.35%), cattle (37.56%), pigs (15.28%), and sheep (8.81%). In addition, in 55.66% of the PPS, bacteria were isolated from food products, in 43.82% from living or slaughtered animals, and in the remaining 0.52% from drag swabs (e.g. fecal samples, eggshells). The platform includes resistance rates for 143 antimicrobials grouped in 37 different families based on their chemical structure^42^. Amongst all the resistance rates available in *resistancebank.org*, 13,163 (59%) are for drug-pathogen combinations recommended for AST by the AGISAR^40^.

### Software implementation

We coded *resistancebank.org* in the open-access R language^43^, in combination with JavaScript and CSS code for the UI (full code^44^ available at https://github.com/hegep-eth/resistancebank.org). We used the functionalities of the *shiny* package^45^ to translate the R code used for the software architecture into HTML language to produce an online platform accessible from all major web browsers: Safari, Google Chrome, Microsoft Edge, Internet Explorer, and Opera. Once completed, we deployed the application on the shinyapps.io servers (https://www.shinyapps.io), a cloud service that, with our configuration, can guarantee simultaneous access to the platform to 2,500 users.

We used the *leaflet* R package to display spatial data^35,46,47^ and add geographic layers in the UI. For displaying the AMR maps, we used a geographic information system software (QGIS 2.18^48^) to produce raster tiles (light square images in.png format) at ten different zoom levels. We stored 2.8 million tiles on a GitHub Pages website linked, as an online resource, to the *leaflet* object used to define the maps. Depending on the zoom and map position, the platform loads just the necessary maps tiles to ensure smooth navigation across zoom levels.

For remote data collection and storage, we used the R packages *rdrop2* and *aws3* to interface the platform with cloud storage services, respectively Dropbox and Amazon Web Services. With every new submission, *reistancebank.org* uploads the corresponding .csv file in an online folder, emptied every time a human operator approves the new submissions. The central database (and its main sub-datasets), the plots present in each pop-up window of the geographic markers, and all of the files downloadable from *resistancebank.org* are stored remotely too. We used functions implemented in packages of the ROpenSci project (e.g. *europepmc*) to gather the bibliographic information of the collected PPS.

Finally, we used a reactive R Markdown document to generate AMR country reports downloadable from *resistancebank.org* in.pdf format. We connected the Markdown file (.Rmd format) to the source code of *resistancebank.org* through an input parameter, i.e., a country name present in the application that depends on a specific object (datasets, functions, etc.). This country name can be specified by the users directly in the UI (e.g., selection of an LMIC through a drop-down menu). Based on that choice, the R Markdown document uses just a specific set of data (or functions) associated with one country to produce its corresponding country report.

## Results

### User interface

**Fig. 2:**
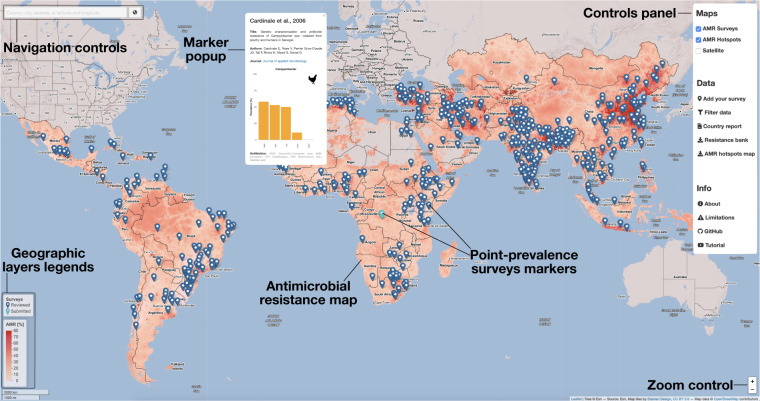
User Interface. Antimicrobial resistance map (red) showing the proportion of antimicrobials with resistance higher than 50% (P50). The point-prevalence surveys are represented as blue geographic markers linked to a pop-up window displaying resistance rates and bibliographic information. The Controls panel enables activating individual geographic layers, downloading the database of point-prevalence surveys, filtering the database, uploading new point-prevalence surveys, and displaying country reports.

The UI of *resistancebank.org* is organized around an interactive map (Fig. 2). The red map shows spatial variations in a summary metric of resistance: P50, the proportion of antimicrobials tested for which bacteria have developed a resistance higher than 50%^26^. This index has been predicted by geospatial models at 10 × 10 km resolution for every LMIC using PPS^26^. Updates of the P50 map will be conducted on an annual basis.

**Fig. 3:**
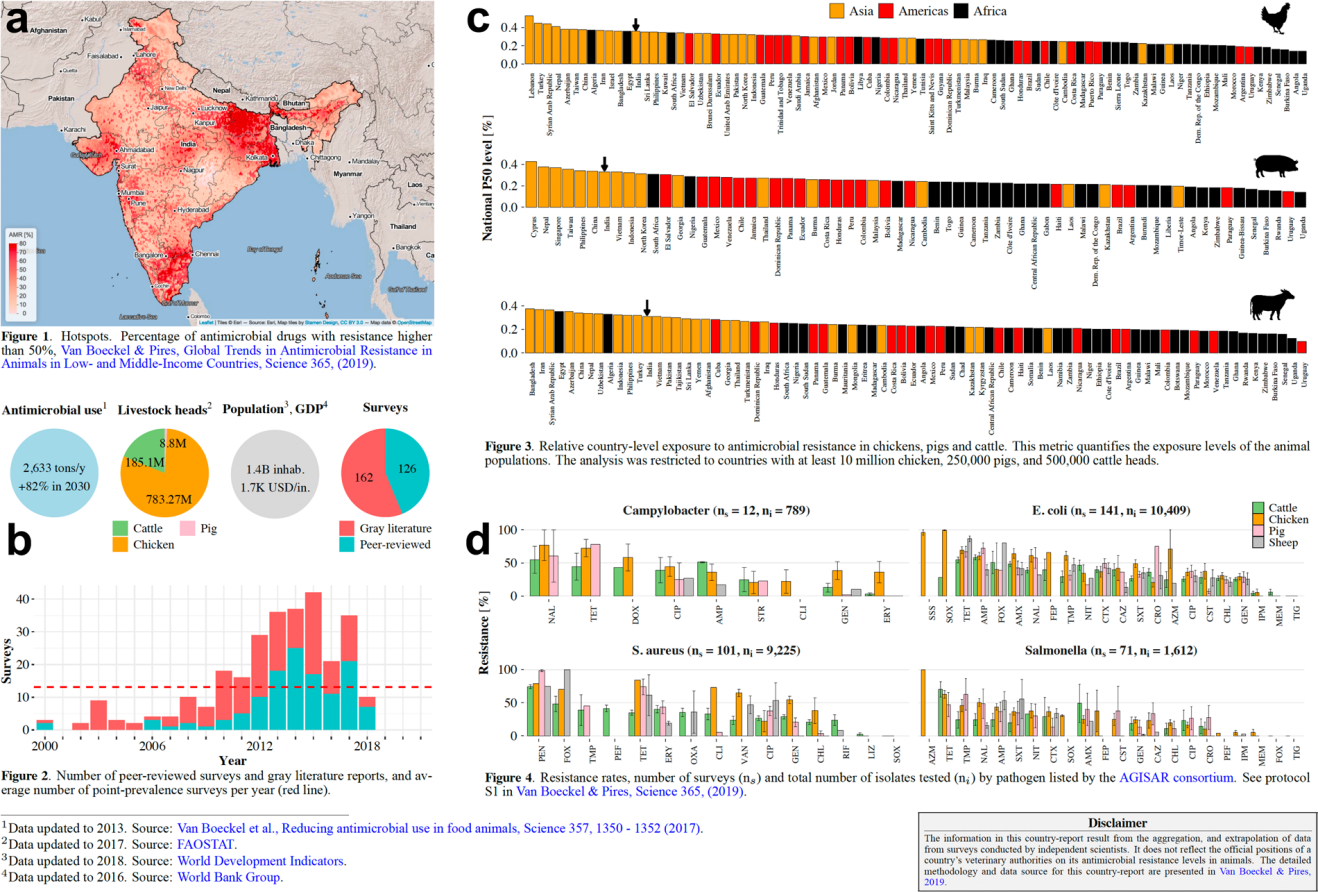
Country report downloadable from resistancebank.org. (**a**) Country-level antimicrobial resistance map. (**b**) Socio-demographic indicators (antimicrobial use and its projected increase in 2030, livestock heads, population, and gross domestic product per inhabitant) and the number of point-prevalence surveys grouped by year and type of paper (peer-review or gray literature). (**c**) Country-level exposure to antimicrobials in chickens, pigs, and cattle (Supplementary Materials of Van Boeckel & Pires, 2019^26^). (**d**) Resistance rates grouped per drug-pathogen combinations listed by the Advisory Group on Integrated Surveillance of Antimicrobial Resistance.

The panel in the top-right corner is divided into three sections: the “Maps” section controls the activation of every geographic layer available on *resistancebank.org* (PPS, AMR map, satellite base map). In the “Data” section, users can download the database of PPS (.csv format) described in Table 1 via the “Resistance bank” button, as well as the P50 raster (.tif format) via the “AMR hotspots map” button. The “Country report” button displays a panel containing a summary of the AMR reports available for each country (Fig. 3). From this panel, it is possible to download the country report for each country where at least one PPS has been reported. Along with the report, users can also download a country-based subset of the central database (.csv format) containing all the data used to generate this output.

Through the “Add your survey” menu, users can choose amongst two modalities for uploading their PPS data in *resistancebank.org*: either by filling an online form, or an Excel template (see next section). The last button in the “Data” section, “Filter data” allows users to filter the database for countries, animal species and animal sample origin, pathogens and if their combination with the antimicrobials aligns with the AGISAR guidelines. In addition, it is also possible to filter the database for an individual antimicrobial class defined by the World Health Organization based on its importance for human medicine^42^ (e.g. 3^rd^ generation cephalosporine). Users can then download the filtered results or display them on the map. In the latter case, when users filter for the antimicrobial class, the geographic marker intensity colour of each survey will vary based on the average resistance rate for the selected antimicrobial class.

Finally, the “Info” panel links to the software’s GitHub repository (https://github.com/hegep-eth/resistancebank.org) and a YouTube video (https://youtu.be/TpMQ_3JLJ2I) illustrating how to use the different sections of the platform.

### Uploading new data

One of the objectives of *resistancebank.org* is to provide up-to-date data visualizations for policymakers continuously collecting evidence from potential users who conducted a PPS in LMICs. To this end, users can input their AST data *on resistancebank.org* using an online form or a pre-filled Excel template. The Excel template can be downloaded and subsequently uploaded from the platform via the “Upload template” button under the “Add your survey” section. Before integrating a new survey, the platform executes automatic verifications for possible misspelled words and typos. If necessary, typos are corrected after comparison with a set of correct words provided for every template field. If *resistancebank.org* recognizes errors that can’t be corrected automatically (e.g., numerical values outside an appropriate range, such as AMR rates higher than 100%), the platform will invite users to revise their inputs. If users wish to use an online form instead of an Excel template, a step-by-step user guide is provided in the next paragraph (“Use Scenario”).

Following the submission of the template or the form, the application automatically performs bibliographic research in the NCBI and PubMed databases to control if the users have provided a valid Digital Object Identifier (DOI) of their study. The application will also automatically extract information on the author(s) name, study title, publication year, journal name, and a link to the journal website associated with the survey submitted. Following submission, a new temporary geographic marker is added on the map, while awaiting further verification by a human operator who is notified of a new submission by email. This marker is light blue, to differentiate it from dark blue markers corresponding to confirmed studies (see Fig. 2 or the YouTube video). The human operator verifies the new data through an internal auxiliary software developed to support *resistancebank.org*. These verifications include a critical interpretation of the resistance rates reported and breakpoints values used for each drug-pathogen combination. The human operator may contact the authors of the study to request corrections/clarifications, and then give its final approval to a submission and merge it with the other surveys in the database. After the upload of a new survey, a near-real-time update of the platform is triggered such as to update all outputs (database available for download and country-level reports). This final step enables *resistancebank.org* to present only the most recent aggregated AMR results based on the PPS available in the scientific literature.

### Use scenario

**Fig. 4:**
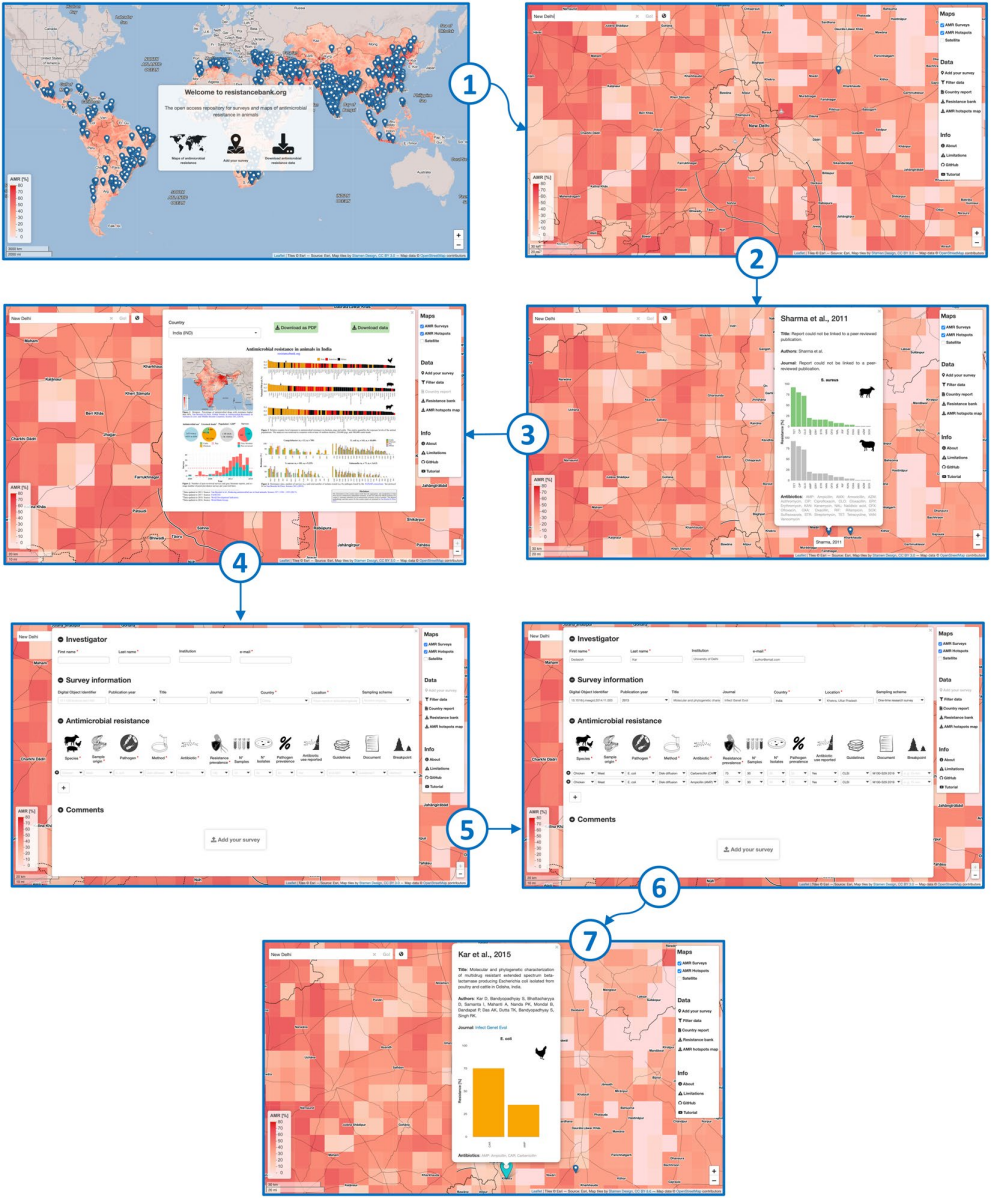
Workflow of the submission of a point-prevalence survey on *resistancebank.org*. Panel 1 shows the home screen of *resistancebank.org*. The steps from 2 to 4 show how the users can explore the visual outputs of the platform, i.e., the antimicrobial resistance map at (10 × 10 kilometers), the resistance rates present in the pop-up window of a point-prevalence survey represented as a geographic marker, and the country report. Panels 5 and 6 show the steps to input new data. Panel 7 shows temporary geographic markers, bar charts, and bibliographic information when the submission of a survey is complete.

We describe examples of the possible use of the platform by a user who wants to upload his/her AMR survey conducted, hypothetically, on a farm near New Delhi, India. The subsequent steps (visually represented in Fig. 4 and the YouTube video) aim to give an overview of the functionalities of *resistancebank.org* and the procedure to submit a new survey.

1. A user launches the online platform by connecting to https://resistancebank.org.
2. The user starts exploring the AMR map and the PPS geographic markers (in the Controls panel section “Maps” these two geospatial layers are both active when the application starts). The user can zoom in on the desired location or type the location name in the navigation bar in the top left part of the UI to explore P50 levels near New Delhi. If the user has recorded the precise coordinates of the study, the input text box can also accept latitude and longitude (separated by a space) in decimal degrees.
3. The map view is now centered in New Delhi. The user can start exploring the P50 levels around the city and the PPS information aggregated at the animal species level present in the geographic markers. Their pop-up window also contains a URL to connect the user to the journal webpage of the study to retrieve additional information besides the ones presented in *resistanbank.org*.
4. Detailed information about the national AMR situation in India is available in the panel accessible through the “Country report” button. Once “India” is selected from the drop-down menu, the country report is ready for download, together with the database of the PPS data collected just in India.
5. The user decides to upload a PPS in *resistancebank.org*. He/she clicks on the “Add your survey” button, a new panel will open, and then the user can decide to upload data through an online form or an Excel template. The hyperlink “.xlsx template” triggers the download of a .zip folder that contains the template and a guide on how to fill it. For this example, we will use the online form.
6. In the upper section of the form, the user can input his contact information. The second section of the form concerns bibliographic information: if a DOI is available, fields as title, journal, and publication year will be automatically filled. In this section, the user must specify the study region, the location (e.g., city, address, or latitude and longitude), and the sampling scheme adopted for the study (if it is a routine, a longitudinal study, a one-time research survey, or a study mandated by public authorities). In the AMR section, every row corresponds to an AST. The mandatory fields are animal species, the animal sample origin (e.g., different swabs, stools, meat, eggshells, gut, dairy products, etc.), the pathogen, the AST method, the antibiotic tested, and the relative value of resistance. Other non-mandatory fields are the number of isolates (at least 10 to be valid for a survey or template submission), the prevalence, the strain used in the quality control of the AST performance, the report of the use of antimicrobials in the farms where animal samples were collected, and the breakpoints. If the researcher tested more than one antibiotic, he/she can add a row that will keep the information from the previous row, except for the new compound name and its resistance value.
7. Once uploaded, the form panel closes automatically, notifying the user of the submission. The light blue geographic marker will appear on the location specified in the form. The geographic marker will remain in a temporary status until a human operator accepts the new submission and merges it with the existing database stored in *resistancebank.org*.

## Discussion

Thus far, a large body of evidence on AMR trends in animals in LMICs was scattered across the veterinary literature. *resistancebank.org* is a starting point to integrate this information. The platform is a surrogate but not a substitute for state-of-the-art systematic surveillance systems^49,50^. The goals of the platform are to summarize current knowledge on AMR in animals and to provide a tool for strengthening its evidence-based surveillance with additional PPS in the future. Furthermore, it overcomes barriers associated with traditional scientific publication (publication fees and access fees), thereby improving the visibility of researchers from LMICs, empowering local communities of scientists, and encouraging national networks coordinators to release their findings onto the website.

Locally, *resistancebank.org* could be used to encourage epidemiological investigations by field officers from LMICs in areas of particular interest. Globally, *resistancebank.org* offers the opportunity to support the actions of international funders such as the Bill & Melinda Gates Foundation, the Fleming Fund, the Food and Agriculture Organization, and the World Organization for Animal Health. In particular, areas identified as hotspots of resistance (P50 > 0.4) could be used to investigate the effects of stewardship campaigns, and alternatives to antimicrobials, such as vaccines and probiotics^51,52^.

Before *resistancebank.org*, different studies have centralized AMR data to describe their large-scale trends both in foodborne and human pathogens^6,26,53,54^. However, the use of an online platform has multiple advantages over individual studies. First, given its open-access nature, downloading and uploading information can be done free by anyone. Second, the diversity of outputs: we provide maps, summary reports for policymakers but also detailed data about resistance rates in individual surveys, with the possibility to filter them at a national- and microbiological-level to better target interventions. Third, the information on *resistancebank.org* is continuously updated in near-real-time. Fourth, the platform provides a much-needed -and thus far missing- focal point of data for a community of researchers studying the epidemiology of AMR in animals. For humans, online platforms that display AMR trends do exist: the Surveillance Atlas of Infectious Diseases^55^ developed by the European Centre for Disease Prevention and Control, and *resistancemap*^56^, developed by the Center for Disease Dynamics, Economics and Policy, display, respectively, European and global yearly AMR trends in common human pathogens. However, unlike *resistancebank.org*, these platforms lack the high spatial resolution of the data, since they aggregate trends at the country-level. While such trends are informative, the granular information underlying them is unfortunately not available in open access. Furthermore, these platforms do not include a possibility for uploading new surveys or dataset by external users. Similar platforms focus on the genetic determinants of AMR^57,58^, and how these affect the spread of pathogens. These include, amongst others, Microreact^59^ and Nextstrain^60^, and are complementary to the phenotypic information provided on *resistancebank.org*. For animals, WHONET^61^ (https://whonet.org) stores users’ AST results obtained from individual bacterial isolates. However, this platform is not currently available for every operating system, and -to the best of our knowledge- does not include geographic information on AMR trends in a centralized context. In contrast, *resistancebank.org* can be used with any internet browser. Our platform has been developed to complement current tools available for AMR surveillance in animal production which requires international attention given its potential implications on human health, animal health, and the long-term sustainability of the livestock sector.

## Limitations

The data presented in *resistancebank.org* come with limitations. The first set of limitations concerns the quality of the event-based surveillance data and comparability across surveys. In the human population, the majority of studies focus on diagnostic samples taken mainly from sick patients. In contrast, in animals, surveillance relies on different data collection contexts: sampling of living versus dead animals, sampling of animal food products, outbreak investigation, sample collection required by food regulatory authorities, etc. These different sampling contexts, which are inherent to event-based surveillance, represent a challenge to the harmonization and the interpretation of resistance rates reported on this platform. In particular, the surveys listed on *resistancebank.org* may differ in terms of i) sampling strategy (random or convenient), ii) animal breeds and farming systems, iii) the number of isolates tested per survey, iv) testing, and v) the degree of aggregation used for reporting antimicrobial rates in each survey (population versus isolate-level information). For these reasons, in *resistancebank.org*, we allow users to specify additional surveys information such as the sampling scheme, the guidelines and breakpoints used for AST, the quality control strains used, etc., to include as much information about these factors that may affect the interpretation of the resistance rates reported.

The second set of limitations concerns the attempt to summarize trends in resistance across drug-pathogen combinations using P50: the proportion of drugs tested in a survey with resistance rates higher than 50%. From a practical perspective, P50 expresses the probability of providing treatments that work out of a portfolio of treatment options, when antimicrobial therapy is indicated for a medical condition. Multiple summary metrics have been proposed^62–65^ and debated^66^ to aggregate resistance rates to multiple drug-pathogen combinations. As with every attempt to reduce this complexity, P50 comes with sources of uncertainty. First, the number of drugs tested in each survey can differ, and this can typically be influenced by the methods used for antimicrobial susceptibility testing in different laboratories (diffusion vs dilution methods), although a good agreement has been shown between the methods^67^. Second, in some surveys, screening for resistance of second-line drugs such as imipenem may be conducted on a subset of the isolates and introduce bias in P50 estimates. In this study, sub-sampling for second-line antimicrobials was limited to 34 out of 1,940 estimates of P50. Third, P50 reflects the number of compounds with resistance higher than 50% rather than the number of classes of compounds with resistance higher than 50%. Therefore, *resistancebank.org* also provides resistance rates for classes of compounds considered medically important by the WHO^42^. The P50 is a summary metric intended to help resource allocation against AMR in countries where systematic surveillance is limited. However, because of the non-systematic nature of the data P50 summarizes, comparisons of resistance rates for individual drug classes should be preferred for informing public health strategies.

The third set of limitations concerns the intensity of our data collection efforts between countries. Our online literature search was supplemented by field officers who collected PPS on paper during visits to veterinary schools. However, these field visits could only be conducted in India, where our collaboration network is extensive. Collaboration with international organizations could help leverage a larger network of field officers to supplement the information currently in *resistanbank.org*. We conducted the literature searches in six languages (English, Mandarin Chinese, Spanish, French, Portuguese, and German). Although these languages are spoken by 46.6% of the world population^68^, further inquiries in other languages could help supplement our database. Finally, the computational cost of re-running the geospatial model is currently preventing instantaneous updates of the AMR map on a global level and should be the focus of future research efforts to move from yearly updates of our maps to daily updates. For the reasons listed above, *resistancebank.org* is an imperfect surrogate to systematic surveillance systems. It is a platform reporting large-scale trends in AMR meant to help international funders to target their efforts in the short term and facilitate the development of a global systematic surveillance system in the long term.

## Data Availability

All data presented in this article are available at https://resistancebank.org.

## Code availability

The scripts and the files of *resistancebank.org* are available in its GitHub repository (https://github.com/hegep-eth/resistancebank.org). We developed the platform through the R software (version 4.0.2) and the RStudio Integrated Development Environment (IDE; version 1.3.1073). In its current version *resistancebank.org* relies on the following dependencies: *aws.s3* (0.3.21), *dplyr* (1.0.2), *europepmc* (0.4), *ggimage* (0.2.8), *ggmap* (3.0.0), *ggplot2* (3.3.2), *gmailr* (1.0.0), *gsubfn* (0.7), *leaflet* (2.0.4.1), *lemon* (0.4.5), *raster* (3.3–13), *rcrossref* (1.0.0), *rdrop2* (0.8.2.1), *rgdal* (1.5–16), *rmarkdown* (1.15), *scales* (1.1.1), *shiny* (1.5.0), *shinyBS* (0.61), *shinyjs* (1.1), *shinyWidgets* (0.5.3), *stringr* (1.4.0), *svglite* (1.2.3.2), *sp* (1.4–5).
